# Metagenomics and In Vitro Growth-Promoting Experiments Revealed the Potential Roles of Mycorrhizal Fungus *Humicolopsis cephalosporioides* and Helper Bacteria in *Cheilotheca humilis* Growth

**DOI:** 10.3390/microorganisms13102387

**Published:** 2025-10-17

**Authors:** Yawei Liu, Yuhao Shang, Xin Wang, Xiao Li, Zhiming Yu, Zhanghui Zeng, Zhehao Chen, Lilin Wang, Taihe Xiang, Xiaoping Huang

**Affiliations:** College of Life and Environmental Sciences, Hangzhou Normal University, Hangzhou 311121, China; 2022210304054@stu.hznu.edu.cn (Y.L.); 2023112010024@stu.hznu.edu.cn (Y.S.); 2022210304049@stu.hznu.edu.cn (X.W.); 2022210301023@stu.hznu.edu.cn (X.L.); yuzhiming@hznu.edu.cn (Z.Y.); zhzeng@hznu.edu.cn (Z.Z.); zhchen@hznu.edu.cn (Z.C.); llwang@hznu.edu.cn (L.W.); xthcn@hznu.edu.cn (T.X.)

**Keywords:** *Cheilotheca humilis*, carbon sources, mycoheterotrophic, mycorrhizal fungus, mycorrhizal help bacteria

## Abstract

In mycorrhizal symbiotic relationships, non-photosynthetic myco-heterotrophic plants are unable to supply photosynthates to their associated fungi. On the contrary, they rely on fungal carbon to sustain their own growth. Mycorrhizal fungi can mediate plant interactions with the rhizosphere microbiome, which contributes to the promotion of plant growth and nutrient uptake. However, the microbial community and key microbial species that function during the growth of the myco-heterotrophic plant *Cheilotheca humilis* remain unclear. In this study, we evaluated the microbial community associated with *Cheilotheca humilis*, which was confirmed via morphological characteristics typical of this plant species. Metagenomic analysis showed that the *Afipia carboxidovorans* was dominant at species level. Based on the LDA score, *Bradyrhizobium ottawaense* exhibited the higher abundance in the CH-B group (related to bud) while *Afipia carboxidovorans* was identified from the CH-F group (related to flower). Microbial co-occurrence networks showed that the *Rhizobium* genus, *Herbaspirillum* genus, and Cyanobacteriota were defined as core functional microbial species. To explore the potential microorganisms, metagenome-assembled genomes (MAGs) of the rhizosphere microbiome identified 14 medium- and high-quality MAGs, mainly involved in carbon fixation, nitrogen transformation, and phosphorus metabolism, possibly providing nutrients for the plant. Furthermore, a total of 67 rhizospheric and 66 endophytic microorganisms were isolated and obtained. In vitro experiments showed that the mycorrhizal helper bacteria (MHBs) *Rhizobium* genus and *Pseudomonas* genus possessed the ability of nitrogen fixation, phosphate solubilization, and siderophores production. Most importantly, the mycorrhizal fungus *Humicolopsis cephalosporioides* was obtained, which could potentially produce cellulase to supply carbohydrates for host. The findings suggest the mycorrhizal fungus *Humicolopsis cephalosporioides* and helper bacteria have great potential in the growth of the myco-heterotrophic plant *Cheilotheca humilis*.

## 1. Introduction

The Monotropoideae subfamily of the Ericaceae consists of 10 genera comprising 12 species [1]. All species in this subfamily are achlorophyllous throughout their entire plant and lack the genes associated with photosynthesis [2,3]. These plants are suspected to acquire fixed carbon from surrounding photosynthetic organisms via mycorrhizal mycelia, which is why they are called myco-heterotrophic plants [4]. Thus, it is of great importance in studies on the evolution of plant–fungal symbiotic interactions.

Certainly, the distribution of these species also exhibited distinct characteristics. For example, myco-heterotrophic plants *Monotropa hypopitys* L. and *Monotropa uniflora* L. were widely distributed throughout the Northern Hemisphere, whereas *Monotropastrum humile* was a monotypic genus found in limited areas of Eastern Asia [1]. Furthermore, the generic classification of *Monotropastrum* was also confusing, which led to *Monotropastrum* often being considered synonymous with *Cheilotheca* [5]. In addition, there is relatively little research on *Cheilotheca*, and the majority of studies have centered on species belonging to the genus *Monotropa*, especially *Monotropa uniflora*.

*M. uniflora*, a close relative of *Monotropastrum*, has attracted significant attentions from researchers. For example, the root systems of *M. uniflora* were characterized using light microscopy and scanning electron microscopy [6]. The mycorrhizal fungi associated with *M. uniflora* differed from those associated with *M. humile* [7]. Notably, myco-heterotrophic plants obtained carbon sources from associated mycorrhizal fungi, either fully or partially [8]. For instance, it has been demonstrated that *Gastrodia confusa* can acquire carbon by parasitizing wood- or litter-decaying fungi [9]. *Cheilotheca humilis,* like *M. uniflora,* is also a fully myco-heterotrophic plant, meaning it relies entirely on fungal symbiosis for carbon acquisition. However, the mycorrhizal fungi that function in the growth of *Cheilotheca humilis* remains unclear so far.

As is well known, the rhizosphere served as a critical zone for plants, with its microbiome being tightly linked to plant growth and development [10]. Rhizosphere soil, together with its microbial community, exerted a significant influence on plant nutrient acquisition [11]. Plants and rhizosphere soil microorganisms engaged in frequent nutrient exchange [12]. Earlier studies primarily focused on identifying microorganisms using ITS and 16S rRNA sequencing, an approach that led to an underestimation of microbial diversity. Currently, metagenomic sequencing is likely to shed clear light on the assembly patterns of microbiomes during the growth of *Cheilotheca humilis*.

With the rapid development of high-throughput sequencing, metagenomics has been extensively used as a powerful tool for investigating the species and functional composition of microbial communities [13]. For example, the result of metagenomics suggested that *Proteobacteria* was the largest taxonomic group in soil and its abundance was significantly increased after Bt cotton cultivation [14]. Metagenomics results showed that *Bacillus altitudinis* LZP02 stimulated some beneficial bacteria, such as *Novosphingobium*, *Acidovorax*, *Sphingomonas*, and *Devosia*, to the rhizosphere [15]. It has been reported that *Paenibacillus* sp. significantly enhanced the seed germination and plant growth of *Astragalus mongholicus* [16]. Furthermore, metagenomic-assembled genomes (MAGs) could uncover potential microbial consortia modulating key growth and development processes. For example, a total of 202 unique MAGs comprising all major taxa present within the *Pyropia haitanensis* microbiome were constructed, involving in-nitrate reduction, taurine metabolism, and auxin synthesis [17]. In drought-stressed rhizospheres, 55 draft MAGs were obtained, and the taxonomic assignment of these MAGs revealed bins belonging to the major root-associated phyla, including Actinobacteria, Proteobacteria, Acidobacteria, and Gemmatimonadetes, among others [18]. Certainly, in addition to the aforementioned microorganisms, mycorrhiza helper bacteria (MHBs) also acted as a third symbiotic partner, thereby affecting the symbiotic relationship between mycorrhizal fungi and plant roots [19]. Nevertheless, there was a deficiency in our knowledge about the microbial community and key microbial species that played potential roles in the growth of *Cheilotheca humilis*.

To address the microorganism community assembly and diversity in the rhizosphere of *Cheilotheca humilis*, metagenomics sequencing was performed. To further mine the uncultivable microorganisms that might play roles, the metagenome-assembled genomes (MAGs) were binned. In addition, in vitro growth-promoting experiments were performed to demonstrate which microorganisms possessed the greatest potential to provide carbon sources and nutrients. In conclusion, these findings provided novel insights into the growth of the myco-heterotrophic plant *Cheilotheca humilis*.

## 2. Materials and Methods

### 2.1. Plant Materials and Morphological Observation

**Figure 1:**
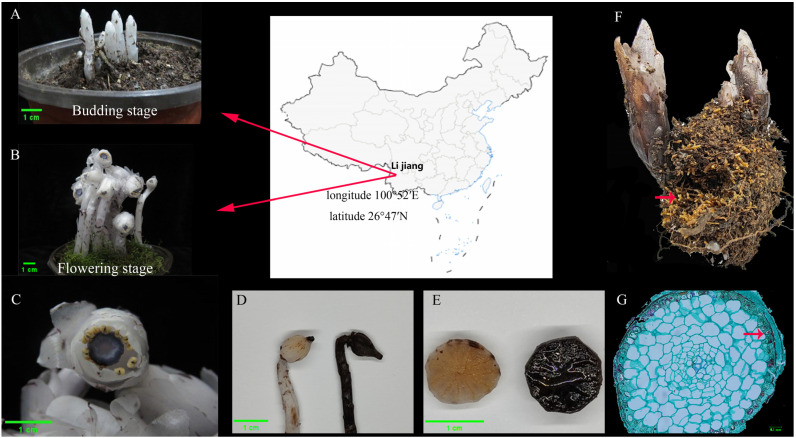
Morphological observation and taxonomic status of the plant in this experiment. (**A**) The sampled plant at the budding stage. (**B**) The sampled plant at the flowering stage. (**C**) Flowers, top view. (**D**) White and blackened swollen ovary. (**E**) Eleven parietal placentation of ovary by transverse section. (**F**) The root ball of *Cheilotheca humilis*. Red Arrowheads indicate root tip apices. (**G**) Paraffin sectioning of root. Red Arrowheads indicate the presence of mycelium and fungal mantle.

Twelve plant materials at budding stage and flowering stage, together with the surrounding soil, were collected from the mountains in Lijiang City (Longitude: 100°52′ E; Latitude: 26°47′ N), Yunnan Province, China (Figure 1). Morphological characteristics of the plant materials were identified. In detail, the plant height, leaf, flower, and ovary of the aboveground part were measured, transected, and observed, respectively. As for the underground part, paraffin section analysis of roots was performed according to the previous method [20]. Briefly, the roots were fixed with FAA solution. After soaking in ethanol with different concentration gradients, the samples were embedded with paraffin and sectioned into 8-μm slices. Subsequently, the sections were dewaxed, dyed, decolorized, and then sealed. Finally, the images were acquired with the digital camera.

### 2.2. Sampling of the Rhizosphere Soil Samples

Rhizosphere soil samples from the *Cheilotheca humilis* at budding stage (named as CH-B) and flowering stage (named as CH-F) were gently collected. After shaking off the loosely bound soil, the roots were immersed in a sterile bottle containing sterilized phosphate-buffered saline (pH 7.4) and washed on a shaking platform for 30 min at 200 rpm. After centrifugation, the precipitated particles were defined as a rhizosphere soil. Soil samples from each stage were set up in triplicate and stored at −80 °C for metagenomics analysis.

### 2.3. DNA Extraction and Metagenomic Sequencing

Total genomic DNA from the rhizosphere soil (CH-B and CH-F) was extracted using a PowerSoil DNA Isolation Kit (MO BIO Laboratories, Carlsbad, CA, USA) following the manufacturer’s protocol. The purity and concentration of extracted soil DNA were assessed through a 0.7% agarose gel electrophoresis and NanoDrop 2000 spectrophotometer (Thermo Fisher, Waltham, MA, USA).

For metagenomic sequencing, a total of 1 µg DNA from each sample was used to construct sequencing libraries using the NEBNext^®^ Ultra^™^ DNA Library Prep Kit (NEB, Ipswich, MA, USA), according to the manufacturer’s instructions. In detail, the index codes were added to attribute sequences to each sample, and then DNA samples were segmented by sonication. After the adenylation of 3′ ends of the DNA fragments, the NEBNext adaptor with a hairpin loop structure was ligated. Next, electrophoresis was used to select DNA fragments of approximately 350 bp size. Then, 3 µL USER enzyme (NEB, USA) was used with size-selected and adaptor-ligated DNA fragments at 37 °C for 15 min, followed by 5 min at 95 °C before PCR. PCR was performed with Phusion High-Fidelity DNA Polymerase, and PCR products were purified with AMPure XP system (Beckman Coulter, Beverly, MA, USA). Finally, library qualities was assessed using the Agilent 2100 Bioanalyzer (Agilent Technologies, Santa Clara, CA, USA), and six metagenomic libraries (CH-B1, CH-B2, CH-B3; CH-F1, CH-F2, and CH-F3) were sequenced in paired-end (2 × 150 bp) mode using Illumina NovaSeq 6000 platform (Illumina, San Diego, CA, USA). All raw data has been submitted to the Sequence Read Archive at NCBI under BioProject PRJNA1143161 with accession number SRR30134408-SRR30134413.

### 2.4. Metagenomic Assembly and Gene Function Annotation

After sequencing, low-quality raw data (length < 50 bp or with a quality value < 20) was removed using FastP. Since no genomic information is available for *Cheilotheca humilis*, the genome of its closely related species *Monotropa hypopitys* (GenBank ID GCA_002855965.1) was selected as the reference genome. To avoid any disturbance, reads mapped to the reference genome were removed by Bowtie2 [21]. The remaining reads from each sample were de novo assembled into contigs using Megahit with default parameters [22]. The open reading frames (ORFs) from each sample were predicted using Prodigal, and the predicted ORFs with a length ≥ 1000 bp were translated into amino acid sequences [23]. Subsequently, all gene sequences with an identity ≥ 0.95 and a coverage ≥ 0.9 were clustered to construct non-redundant gene catalogs by CD-HIT [24]. Abundance profiles of genes were estimated by aligning the reads to the gene catalog using SOAP aligner [25]. Functional annotation was performed with DIAMOND software (v2.0.7) against the NR database, Clusters of Orthologous Groups of proteins (COGs) database [26], Kyoto Encyclopedia of Genes and Genomes (KEGG) database [27], and carbohydrate-active enzyme (CAZY) database [28], with the Blastp parameter and an E-value of ≤1 × 10^−5^ [29].

### 2.5. Metagenome Binning, Taxonomy Assignment, and Phylogenetic Analysis

The assembled contigs with length > 1000 bp were binned using MetaBAT2 v2.12.1 [30], MaxBin2 v2.2.4 [31], and CONCOCT [32] with default parameters. The original bins were consolidated and improved with the Bin_refinement modules in MetaWRAP [33]. Metagenome-assembled genomes (MAGs) with medium quality (70% < completeness < 90% and contamination < 10%) and high quality (completeness > 90% and contamination < 10%) were kept for subsequent analysis [34]. CoverM (https://github.com/wwood/CoverM, accessed on 14 March 2023) genome model was employed to calculate the relative abundance of each MAG. Taxonomic assignment of MAGs was achieved using the Genome Taxonomy Database (GTDB) by GTDB-Tk (Version 1.3.0) [35] with the database release207_v2 [36]. Gene function prediction of MAGs was executed using the MetaWRAP-Annotate_bins module with Prokka [37]. The protein-coding sequences were searched by BlastP against the NR, KEGG, COG, Uniport, and CAZY database. Moreover, the genes of MAGs were searched against the CCycDB (https://ccycdb.github.io/, accessed on 19 March 2023), NCycDB (https://github.com/qichao1984/NCyc, accessed on 19 March 2023), PCycDB (https://github.com/ZengJiaxiong/Phosphorus-cycling-database, accessed on 19 March 2023), and GO databases (E-value ≤ 1 × 10^−4^) for functional annotation. The marker genes of carbon cycling gene (CCG), nitrogen cycling gene (NCG), and phosphorus cycling gene (PCG) were filtered (identity ≥ 95%, coverage ≥ 90%). A phylogenetic tree of MAGs was constructed using a concatenated alignment of marker genes in GTDB-Tk with the default parameters (Version 2.1.0). The tree was visualized by the online Interactive Tree of Life platform (iTOL) [35].

### 2.6. Statistical Analysis and Visualization

Statistical analyses were performed using two-tailed Student’s *t*-test via GraphPad Prism version 8 (GraphPad Software Inc., San Diego, CA, USA). Data was expressed as means ± standard deviation (SD). Statistical significance was defined as *p* < 0.05 (*) and *p* < 0.01 (**). Alpha and beta diversity of the microbiome between the samples were calculated with the “vegan” [38] and visualized with the “ggplot2” package [39]. Bray–Curtis distance was analyzed based on permutational multivariate analysis of variance, and the dissimilarities between samples were computed with analysis of similarities. The significant differences between samples were determined by Linear Discriminant Analysis Effect Size (LEfSe) analysis using Wilcoxon tests (*p* value < 0.05 and LDA score > 4).

To explore the correlations among all microorganisms, co-occurrence networks were constructed based on Spearman’s correlation coefficients (|r| > 0.7 and *p*-adjusted value < 0.05) using the R package (v4.1.0). Gephi software (version 0.9.2) was employed to compute topological parameters of the networks, as previously described [40], such as the number of nodes, edges, degrees, and clustering coefficients. Nodes with a degree ≥ 30 were considered as keystone nodes [41]. The stability of the network was evaluated based on the proportion of negative or positive correlations and its modularity [42]. The visualization of the networks was carried out using Cytoscape v3.8.0 [43].

### 2.7. Isolation and Identification of Microorganisms

For the isolation of microorganisms from the rhizosphere soil, soil suspension was serially diluted. The diluent was streaked onto Potato Dextrose Agar (PDA) medium for fungi and Luria Broth (LB) for bacteria. For the isolation of endophytes from the roots of *Cheilotheca humilis*, the roots were first disinfected with 0.1% HgCl_2_ and transversely cut into segments using a sterile knife, and the root segments were subsequently placed on PDA and LB medium. After incubation at 28 °C for three days in the constant temperature incubator, hundreds of microorganism strains were selected for purification based on morphological characteristics. Total genomic DNA was extracted by the Ezup column bacterium and fungal genomic DNA purification kit (Sangon Biotechnology Co., Ltd., Shanghai, China). The universal primers 27F and 1492R were used to amplify rRNA gene of bacteria, while primers ITS1 and ITS4 were for internal transcribed spacer regions of fungi. The PCR products were sequenced, and the resulting DNA sequences were used to identify the genus level of microorganisms against the NCBI GenBank database.

### 2.8. Assay for In Vitro Growth-Promoting Characteristics of Isolated Microorganisms

All isolated microorganisms were selected to evaluate growth-promoting characteristics [44]. In detail, siderophore production was carried out by quantitative assay, as previously described [45], using CAS reagent [46]; the formation of an orange-yellow halo around the microorganism growth indicated the production of siderophores. Ashby nitrogen-free medium, NBRIP medium, and carboxymethyl cellulose selective medium were used to screen isolates with nitrogen fixation, phosphate solubilization, and cellulase production. Isolates were spot-plated on the media and incubated for 3–5 days at 28 °C. The clear halo formation around the colony showed the capability of the isolates [47,48]. The uninoculated media was set as control. Furthermore, the mycorrhizal fungus with cellulase enzyme activity was inoculated onto tobacco hairy roots to investigate the growth promoting effects; additionally, it was sequenced by whole-genome sequencing [49].

## 3. Results

### 3.1. Morphological Observation of Myco-Heterotrophic Plants

The myco-heterotrophic plants were separately sampled from western China during the budding stage and flowering stage. The whole plant was white in color and with an absence of hairs through visual observation (Figure 1A,B). The leaves were scaly, alternate, and broadly ovate in shape (Figure 1B). In addition, it had 3–5 petals and wide strip-shaped and long hairs on the inner surface (Figure 1C). The plant had 12 stamens of equal length, and anther were orange-yellow and covered with small warts. The filaments were slightly flattened with sparse coarse hairs. The stigma was hairless, blue-black, broad, and funnel-shaped with sparse long hair (Figure 1C). After flowering and pollination, the ovary will expand and become rounded, but it will not stand upright, and it maintained a lowered position (Figure 1D). Once detached or damaged, the plant will gradually turn black and spread throughout the entire plant (Figure 1D).

Furthermore, the cross-sectional of the ovary showed that it had 11 parietal placentation (Figure 1E). Based on the abovementioned results, the plant was identified as *Cheilotheca humilis* in taxonomy [50]. More interestingly, the *Cheilotheca humilis* had a root ball and relatively distinct root tips, which were unified with the surrounding soil (Figure 1F). Paraffin sectioning of roots indicated the existence of the fungal mantle and Hartig net (Figure 1G).

### 3.2. The Diversity, Composition, and Structure of Rhizosphere Microorganisms in the Cheilotheca humilis

**Figure 2:**
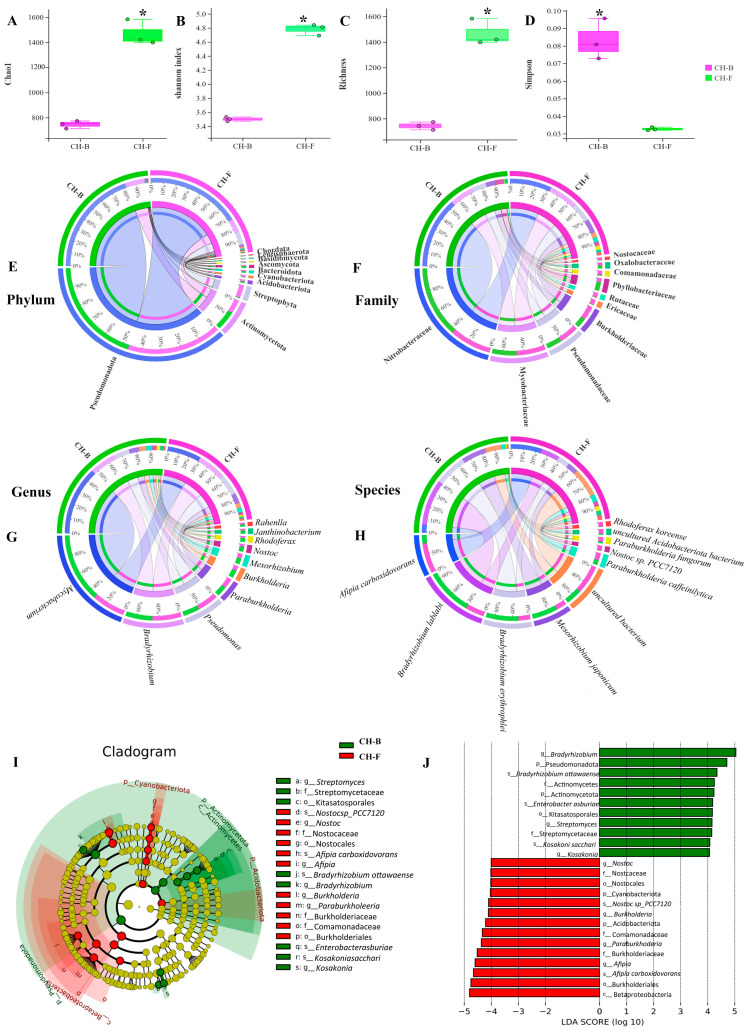
The diversity, composition, and microbial structure of *Cheilotheca humilis* rhizosphere. (**A**–**D**) The alpha diversity Chao1, Shannon, Simpson, and Richness. Statistical significance was defined as *p* < 0.05 (*). (**E**–**H**) The relative abundance of phylum, family, genus, and species level in *Cheilotheca humilis* rhizosphere. (**I**) Top 30 LEfSe circular evolutionary branching graphs. The phylum and class enriched in the CH-F were highlighted with a red shadow and in the CH-B with a green shadow. Red nodes represent taxa enriched in the CH-F at the order/family/genus/species level, while green nodes represent taxa enriched in the CH-B. The yellow nodes denote taxa with no statistical differences. (**J**) Histogram of LDA value distribution of the first 30 LEfSe LDA values at different levels. The greater the LDA value, the more significant its contribution to the differences between the groups is.

After filtering adaptors and low-quality reads, a total of 61,015,573,153 bp clean reads were generated from *Cheilotheca humilis* samples in this study. The metagenomics result of CH-B and CH-F samples showed the presence of 48 phyla, 461 families, 844 genera, and 1588 species. The results of alpha diversity showed that the Chao1, Shannon, and richness of the CH-F group were significantly higher than those of the CH-B group (*p* < 0.05), although the Simpson index showed the opposite trends (Figure 2A–D). At the phylum level, Pseudomonadota was the most abundant phylum in both CH-F and CH-B, followed by Actinomycetota (Figure 2E; Supplementary Table S1). At the family level, Nitrobacteraceae, Mycobacteriaceae, and Pseudomonadaceae were the top three dominant families (Figure 2F; Supplementary Table S1). At the genus level, *Bradyrhizobium* was the most dominant genus, followed by *Mycobacterium*, *Pseudomonas*, *Paraburkholderia*, and *Mesorhizobium* (Figure 2G; Supplementary Table S1). At the species level, *Afipia carboxidovorans*, *Bradyrhizobium lablabi*, *Bradyrhizobium erythrophlei*, and *Mesorhizobium japonicum* were the dominant species (Figure 2H; Supplementary Table S1).

Considering the absence of inter-group discrimination analysis, the LEfSe method was employed to examine differences in abundance between phylum, class, order, family, genus, and species across the two groups. A total of 19 biomarker taxa were identified, comprising 11 taxa in the CH-F group and 8 taxa in the CH-B group (Figure 2I). At the species level, *Bradyrhizobium ottawaense*, *Enterobacter asburiae*, and *Kosakonia sacchari* exhibited higher abundance in the CH-B group based on the LDA score (log10) > 4. In the CH-F group, *Nostoc* sp. *PCC7120* (with the nitrogen-fixation function) and *Afipia carboxidovorans* (with the carbon-fixation function) showed significant differences, implying their pivotal roles (Figure 2J).

### 3.3. Predictive Core Taxa of Rhizosphere Microbial Community in the Mycoheterotrophic Plant Cheilotheca humilis

**Figure 3:**
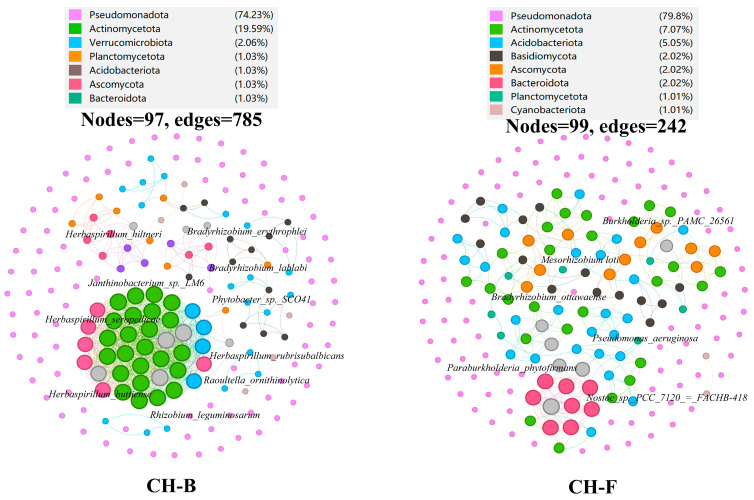
Core taxa of rhizosphere microbial community in the *Cheilotheca humilis*. Co-occurrence network analysis diagram of the rhizosphere microbes of *Cheilotheca humilis* at the species level. Nodes were colored by microbial genera, as explained in the legend. The gray circles represented unassigned. Node size represented the degree, and the thickness of the line between nodes indicated the size of the correlation coefficient between them. The edge represented Spearman’s correlation coefficient (R > 0.6, *p* < 0.05). The networks colored by taxonomy were visualized using the software Gephi (version 0.9.2).

To determine the effect of different plant development stages on microbial co-occurrence patterns, networks were constructed based on Spearman’s correlation coefficient. The core taxonomic group of the microbial community formed a rhizosphere microbial network, consisting of 97 nodes and 785 edges in the CH-B group, while having 99 nodes and 242 edges in the CH-F group (Figure 3). The average path length between all node pairs were 1.22 and 1.34, with an average weight degree of 4.046 and 1.222 and an average clustering coefficient of 0.209 and 0.191, respectively.

According to the network connectivity statistics (degree, closeness centrality, and abundance), the *Herbaspirillum* genus (*H. rubrisubalbicans*, *H. seropedicae*, *H. huttiense*, and *H. hiltneri*) and *Rhizobium* genus (*R. leguminosarum*, *B. erythrophlei*, and *B. lablabi*) were defined as core functional rhizosphere microbial species in the CH-B group (Figure 3; Supplementary Table S2), all belonging to the phylum Pseudomonadota.

In the CH-F group, *Mesorhizobium loti*, *Bradyrhizobium ottawaense*, and *Paraburkholderia phytofirmans* were defined as core functional rhizosphere microbial genera belonging to the phylum Pseudomonadota, while *Nostoc* sp. PCC 7120=FACHB-418 belonged to the phylum Cyanobacteriota (Figure 3; Supplementary Table S2).

### 3.4. Functional Characteristics Analysis of Genes Associated with Rhizosphere Microbes in the Myco-Heterotrophic Plant Cheilotheca humilis

**Figure 4:**
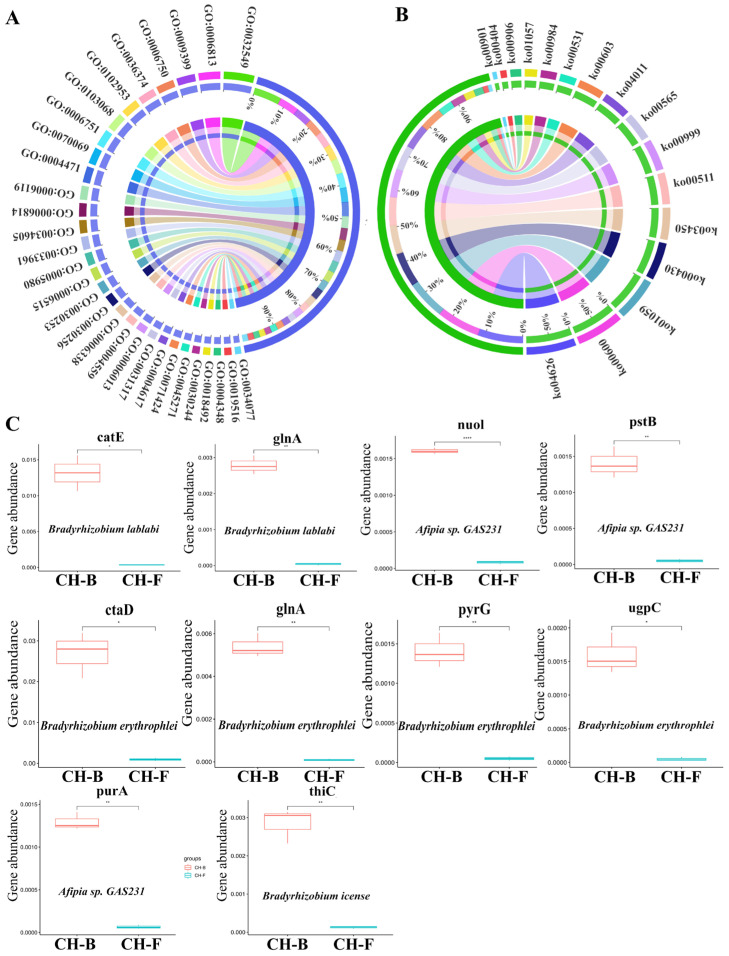
Functional analysis and abundance of genes associated with *Cheilotheca humilis* rhizosphere metagenome. Left circle represents GO functional analysis (**A**), right circle represents KEGG functional analysis (**B**). Functional genes with significant down-regulation of abundance are based on two-side t-tested (* *p* < 0.05, ** *p* < 0.01, **** *p* < 0.0001). Error bars represented standard deviations (**C**).

The predicted protein coding genes were aligned to the GO and KEGG databases for functional classification. At the GO category level, ribonucleoside binding (GO:0032549), nitrogen fixation (GO:0009399), the glutathione biosynthetic process (GO:0006750), hypoglycin A gamma-glutamyl transpeptidase activity (GO:0102953), and the butanediol metabolic process (GO:0034077) were enriched (Figure 4A; Supplementary Table S3). In addition, the plant–pathogen interaction (ko04626), sphingolipid metabolism (ko00600), biosynthesis of enediyne antibiotics (ko01059), taurine and hypotaurine metabolism (ko00430), and indole alkaloid biosynthesis (ko00901) were dominant pathways in the KEGG category level (Figure 4B; Supplementary Table S3).

Furthermore, functional genes abundance and its occurrence in different taxa were identified. Interestingly, a total of 13 functional DE CCGs corresponding to four differential taxa (*Bradyrhizobium icense*, *Afipia* sp. *GAS231*, *Bradyrhizobium erythrophlei*, and *Bradyrhizobium lablabi*), DE NCG glnA corresponding to two differential taxa (*Bradyrhizobium erythrophlei* and *Bradyrhizobium lablabi*), and four DE PCGs corresponding to two differential taxa (*Afipia* sp. *GAS231* and *Bradyrhizobium erythrophlei*) were searched by blast against the CCycDB, NCycDB, and PCycDB (Supplementary Table S4). The abundance of functional genes corresponding to taxa were also displayed (Figure 4C). The functional genes associated with the *Cheilotheca humilis* rhizosphere microbiome might imply the roles of rhizosphere microorganisms in the growth of host plants.

### 3.5. Metagenome-Assembled Genomes (MAGs) in the Rhizosphere of Myco-Heterotrophic Plant Cheilotheca humilis

**Figure 5:**
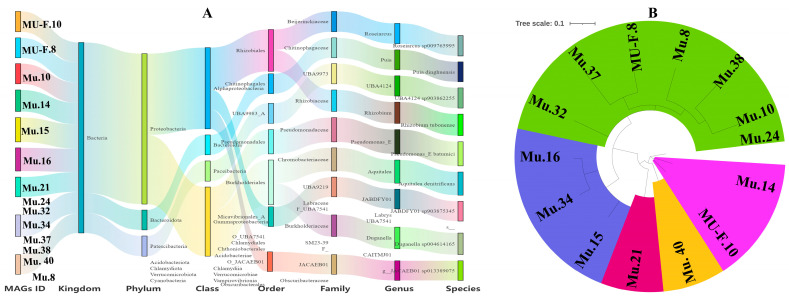
The characteristics of 14 metagenome-assembled genomes (MAGs). (**A**) The 14 MAG taxonomies across different levels. Sankey plot depicts the detailed classifications of MAGs at seven phylogenetic levels (kingdom, phylum, class, order, family, genus, and species). (**B**) Phylogenetic tree of 14 medium and high-quality MAGs. Stars indicate the MAGs with completeness greater than 90%.

To further examine the genetic potential of the rhizosphere microbiome in the *Cheilotheca humilis*, a comprehensive analysis of clean metagenomic data was conducted. After sequence assembly, a total of 55 metagenome-assembled genomes (MAGs) were reconstructed (Supplementary Table S5). Based on a quality assessment, 14 MAGs with 6 medium-quality MAGs (70% < completeness < 90%, contamination < 10%) and 8 high-quality MAGs (completeness > 90% and contamination < 10%) were obtained, which were further classified by mapping against the Genome Taxonomy Database (GTDB) (Table 1). The genome size of each recovered MAG was 804,524 bp to 8,226,318 bp, and the GC content was 41.84–63.76% (Table 1). By ANI analysis and GTDB assignment, the microbial compositions of 14 MAGs at seven different phylogenetic levels were illustrated, with Proteobacteria having the most phylum (Figure 5A). In detail, these MAGs were identified as *Roseiarcus* sp. 009765995 (MU-F.10, completeness = 74.25%; contamination = 6.28%), *Puia dinghuensis* (MU-F.8, completeness = 91.01%; contamination = 7.44%), *UBA4124* sp. 903862255 (Mu.10, completeness = 81.24%; contamination = 0%), *Rhizobium tubonense* (Mu.14, completeness = 89.81%; contamination = 3.27%), *Pseudomonas batumici* (Mu.15, completeness = 75.44%; contamination = 8.77%), *Aquitalea denitrificans* (Mu.16, completeness = 97.86%; contamination = 0.43%), *JABDFY01* sp. 903875345 (Mu.21, completeness = 96.4%; contamination = 2.94%), *Duganella* sp. 004614165 (Mu.34, completeness = 78.06%; contamination = 7.64%), *JACAEB01* sp. 013389075 (Mu.40, completeness = 99.13%; contamination = 1.16%), and another five MAGs belonging to novel species (Table 1; Figure 5B).

### 3.6. Carbon Fixation Pathways in the Rhizosphere Metagenome of Myco-Heterotrophic Plant Cheilotheca humilis

Carbon source was the critical substance for maintaining the normal growth of the plant. For 14 reconstructed MAGs, a total of 476 CCGs involved in-carbon fixation, carbon release, organic biosynthesis, organic degradation, organic transformation, and transporters modules were identified by blast search against CCycDB (Supplementary Table S6).

**Figure 6:**
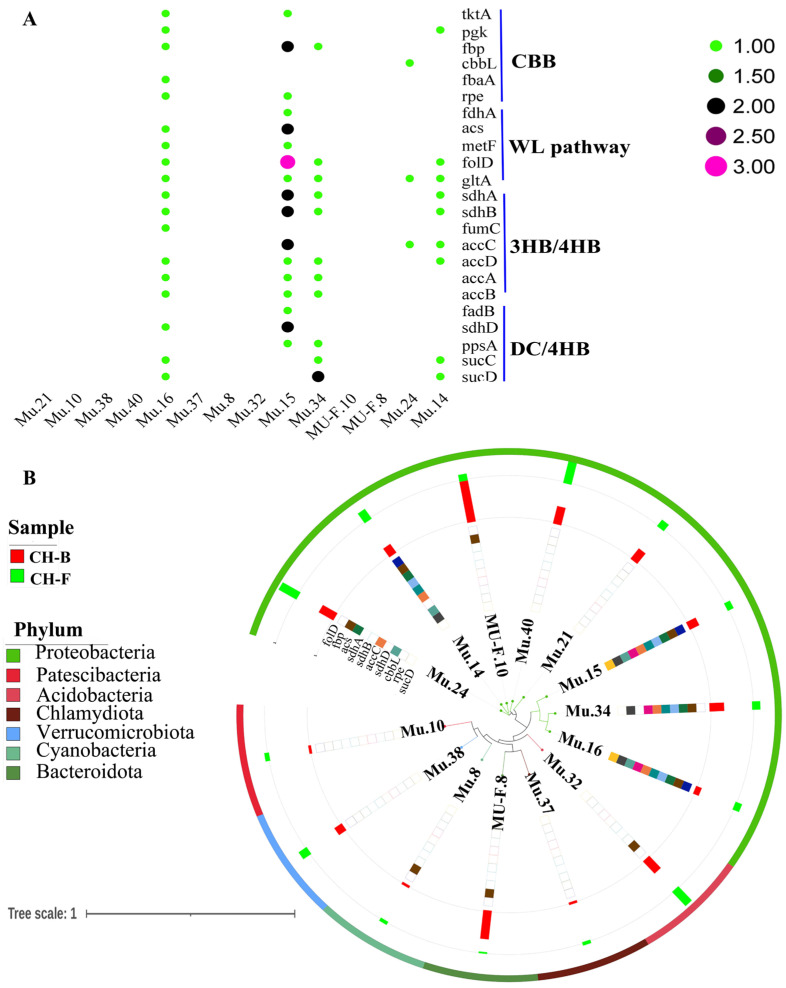
Carbon fixation pathways in the rhizosphere metagenome of *Cheilotheca humilis.* (**A**) The carbon fixation pathway and related genes. (**B**) Phylogenetic tree of gene abundance in 14 MAGs.

Furthermore, the myco-heterotrophic plant *Cheilotheca humilis* had no photosynthetic ability. A bold speculation was proposed that carbohydrates of the host plant were derived from the carbon fixation pathway of microorganisms. Interestingly, six CCGs (*tktA*, *pgk*, *fbp*, *cbbL*, *fbaA*, and *rpe*) involved in the Calvin–Benson–Bassham cycle (CBB), five CCGs (*fdhA*, *acs*, *metF*, *folD*, and *gltA*) involved in the reductive acetyl-CoA pathway (WL), seven CCGs (*accABCD*, *fumC*, *sdhA*, and *sdhB*) involved in the 3-hydroxypropionate bicycle (3HB/4HB), and five CCGs (*fadB*, *sdhD*, *ppsA*, *sucC*, and *sucD*) involved in the dicarboxylate/4-hydroxybutyrate cycle (DC/4HB) were identified (Figure 6A; Supplementary Table S7). Especially, Mu.14 (*Rhizobium tubonense*) and Mu.15 (*Pseudomonas batumici*) contained the top two CCGs with a higher relative abundance at the phylum level (Figure 6B).

### 3.7. Nitrogen-Transforming Network in the Rhizosphere Metagenome of Myco-Heterotrophic Plant Cheilotheca humilis

**Figure 7:**
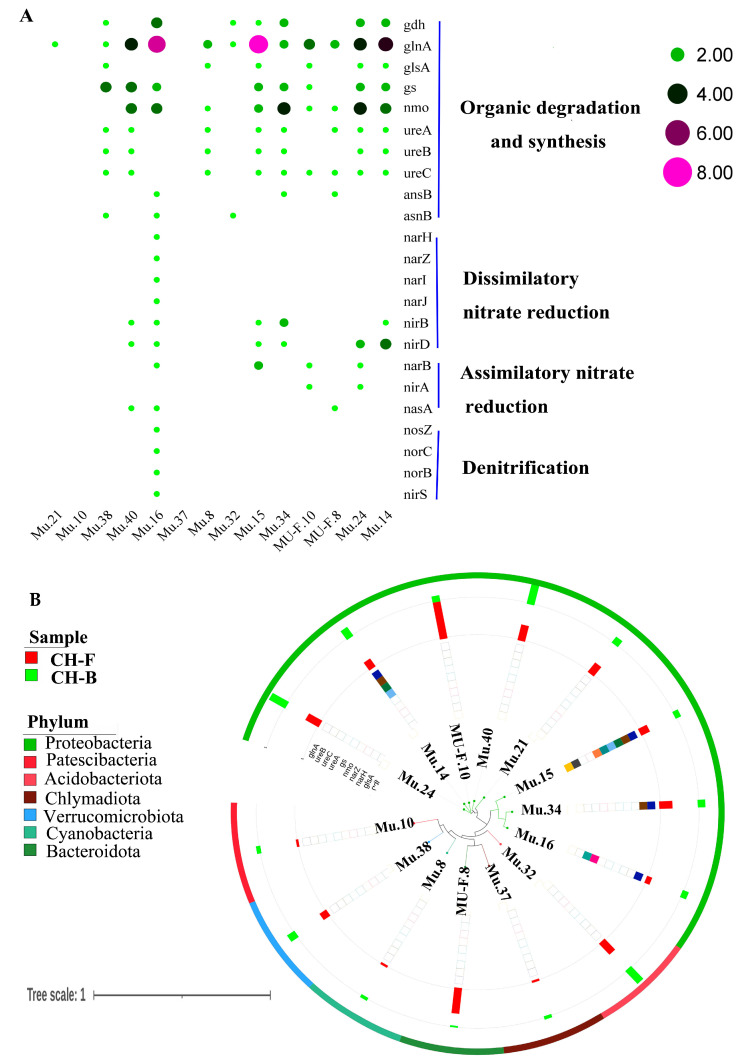
Nitrogen-transforming network in the rhizosphere metagenome of *Cheilotheca humilis*. (**A**) The nitrogen-related pathway and genes. (**B**) Phylogenetic tree of gene abundance in 14 MAGs.

The nitrogen (N) cycle was an important collection of biogeochemical pathways, which received widespread attention in biology research. In particular, four nif genes (*nifD*, *nifH*, *nifK*, and *nifW*) were identified from raw sequencing reads. For 14 reconstructed MAGs, a total of 23 NCGs were identified by blast search against NCycDB, including 10 NCGs (*gdh*, *glnA*, *glsA*, *gs*, *nmo*, *ureA*, *ureB*, *ureC*, *ansB*, and *asnB*) involved in organic degradation and synthesis, 6 NCGs (*narJ*, *narH*, *narI*, *narZ*, *nirB,* and *nirD*) involved in dissimilatory nitrate reduction, 3 NCGs (*narB*, *nirA*, and *nasA*) involved in assimilatory nitrate reduction, and 4 NCGs (*nosZ*, *norB*, *norC*, and *nirS*) involved in denitrification (Figure 7A; Supplementary Table S8). Similarly, Mu.14 (*Rhizobium tubonense*) and Mu.15 (*Pseudomonas batumici*) contained the top two NCGs with a higher relative abundance at the phylum level (Figure 7B).

### 3.8. Phosphorus Metabolism in the Rhizosphere Metagenome of Myco-Heterotrophic Plant Cheilotheca humilis

**Figure 8:**
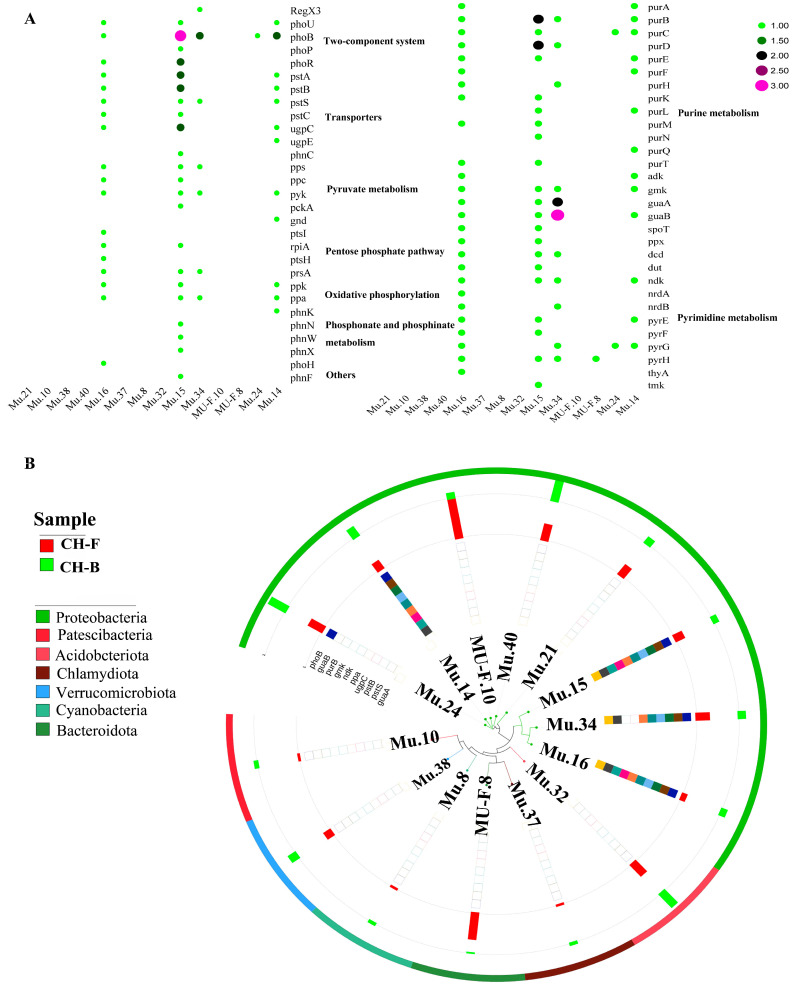
Phosphorus metabolism pathways in the rhizosphere metagenome of *Cheilotheca humilis.* (**A**) Phosphorus metabolism pathway and genes. (**B**) Phylogenetic tree of gene abundance in 14 MAGs.

Notoriously, phosphorus uptake and microbially mediated phosphorus cycling in soil may alter plant growth. For 14 reconstructed MAGs, a total of 59 PCGs were identified by blast search against PCycDB, including 5 PCGs (*RegX3*, *phoB*, *phoP*, *phoR*, and *phoU*) involved in the two-component system, 7 PCGs (*pstABC*, *pstS*, *phnC*, *ugpC*, and *ugpE*) involved in transporters, 4 PCGs (*ppc*, *pps*, *pckA*, and *pyk*) involved in the pyruvate metabolism, 5 PCGs (*gnd*, *ptsI*, *ptsH*, *prsA*, and *rpiA*) involved in the pentose phosphate pathway, 2 PCGs (*ppa*, *ppk*) involved in oxidative phosphorylation, 4 PCGs (*phnK*, *phnN*, *phnW*, and *phnX*) involved in phosphonate and phosphinate metabolism, 19 PCGs (*purABCDEFHKLMNQT*, *adk*, *gmk*, *guaA*, *guaB*, *spoT,* and *ppx*) involved in purine metabolism, 11 PCGs (*dcd*, *dut*, *ndk*, *nrdA*, *nrdB*, *pyrEFGH*, *thyA*, and *tmk*) involved in pyrimidine metabolism, and 2 other PCGs, *phoH* and *phnF* (Figure 8A; Supplementary Table S9). Similarly, Mu.14 (*Rhizobium tubonense*) and Mu.15 (*Pseudomonas batumici*) contained the top two PCGs with a higher relative abundance at the phylum level (Figure 8B).

### 3.9. Evaluation of the In Vitro Growth-Promoting Ability of Isolated Microorganisms

**Figure 9:**
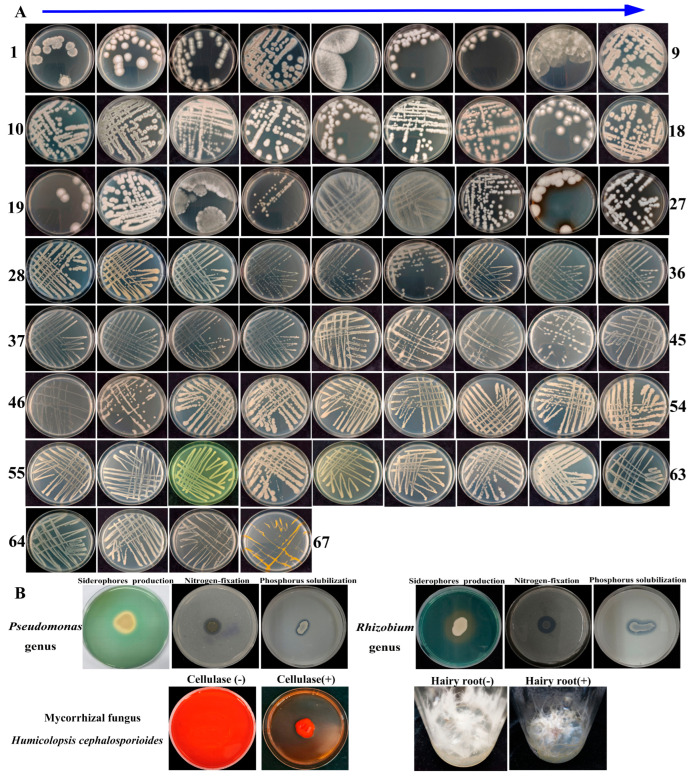
The isolation of rhizospheric microorganisms and growth-promoting characteristics. (**A**) The 67 rhizospheric microorganisms. (**B**) The growth-promoting characteristics of *Pseudomonas* and *Rhizobium* genera, the cellulase-production capacity and growth-promoting effect of *Humicolopsis cephalosporioides*.

To obtain the possible essential culturable microorganisms supporting the growth of the host plant *Cheilotheca humilis*, an extensive isolation procedure was performed. In this study, the 67 rhizospheric (Figure 9A) and 66 endophytic microorganisms (Supplementary Figure S1) were obtained and purified; all isolated microorganisms were deposited in the GenBank database (Supplementary Table S10). In addition, in vitro growth-promoting experiments suggested that the rhizospheric *Rhizobium* genus and *Pseudomonas* genus possessed the ability of nitrogen fixation, phosphate solubilization, and siderophores production (Figure 9B). Most importantly, the result of whole-genome sequencing showed that the isolated fungus 1.1-7 was identified to be mycorrhizal fungus *Humicolopsis cephalosporioides* (*H. cephalosporioides*). The fungus genome data was deposited in the NCBI database under Bioproject: PRJNA1262328 with accession number: JBPDIK000000000. In addition, the mycorrhizal fungus *H. cephalosporioides* can produce cellulase to provide potential carbohydrates (Figure 9B). Due to the limitations in tissue culture technology and the difficult survival characteristics of the plant *Cheilotheca humilis*, the in vivo growth-promoting experiments on the host plant by inoculated microorganisms were not successful, as well as in the hairy root (Figure 9B). Although, the result still provided a novel insight into the survival strategies of the myco-heterotrophic plant *Cheilotheca humilis*.

## 4. Discussion

Most plants acquired carbon sources through photosynthesis. In contrast, certain plants obtained carbon sources from other organisms. These two distinct life strategies were termed “autotrophy” and “heterotrophy,” respectively. Interestingly, “Mycoheterotrophy” described a plant’s capacity to obtain carbon sources from fungi [4]. Prior research on mycoheterotrophy primarily concentrated on physiological ecology [51,52] and the evolution of the chloroplast genome [53,54,55]. Nevertheless, there was a deficiency in our knowledge about the microbial community and key microbial species that played crucial roles in the growth of the *Cheilotheca humilis*. To address this question, metagenomics sequencing was performed in this study.

### 4.1. Metagenomics and Microbial Cultivation Demonstrated the Community Diversity and Key Roles of Mycorrhizal Fungus H. cephalosporioides

Advances in metagenomics revolutionized the understanding of taxonomic profiling and rhizosphere microbial communities. For example, the metagenomics results showed that 30 rhizosphere and 14 root bacterial genera were significantly affected by the CL as an infection [56]. It was reported that the abundance of *Pseudomonas* significantly enriched in the root and rhizosphere of soybean, especially the *Pseudomonas* mutant, could enhance a plant’s salt tolerance [57]. In this study, a total of 48 phyla, 461 families, 844 genera, and 1588 species were identified by metagenomics sequencing. Among them, Pseudomonadota, Nitrobacteraceae, and Bradyrhizobium were predominant (Figure 2E–G; Supplementary Table S1). To obtain the microorganisms identified in the metagenomics, an extensive isolation and cultivation procedure was performed. In total, 67 rhizospheric (Figure 9A) and 66 endophytic microorganisms (Supplementary Figure S1) were obtained. Groundbreakingly, it was the first time in the world that the existence of the specific mycorrhizal fungus *H. cephalosporioides* 1.1-7 in the *Cheilotheca humilis* was reported (Supplementary Table S10).

Considering the host plant and distinctive symbiotic structures, mycorrhizal types were mainly classified into arbuscular mycorrhizas, ectomycorrhizas, orchid mycorrhizas, and ericoid mycorrhizas [58]. Each type had a unique evolutionary background, anatomical features, and ecological traits, consequently exerting diverse effects on plant nutrient uptake and cycling [59]. Especially, ectomycorrhizas fungi formed a Hartig net in which minerals and nutrient materials were exchanged [60]. In this study, paraffin sectioning of the roots showed the existence of the fungal mantle and Hartig net, indicating the specificity of ectomycorrhizas in the plant *Cheilotheca humilis* (Figure 1G). In addition, numerous lineages of ectomycorrhizal fungi had experienced the loss of genes encoding lignocellulose-degrading enzymes [61]. Surprisingly, the mycorrhizal fungus *H. cephalosporioides* displayed a potent cellulase production capacity capable of degrading cellulose in an in vitro experiment, implying this fungi presence could potentially explain part of the mechanisms used by the plant to obtain carbon (Figure 9B).

### 4.2. Mycorrhizal Helper Bacteria Might Involve in the Growth of Cheilotheca humilis by Carbon Fixation Pathway

Generally, mycorrhizal fungi were considered to facilitate plant growth and nutrient uptake with mycorrhizal helper bacteria (MHBs), a most extensively studied mycorrhizal microbiome [62,63,64]. It had been reported that *aerobic carboxydotrophic* bacteria can oxidize carbon monoxide (CO) into carbon dioxide (CO_2_), which is subsequently used for biomass formation via the Calvin–Benson–Bassham (CBB) cycle [65]. *Cyanobacteria* have been proposed as an ideal photoautotrophic host for sustainable bioproduction, which can drive CO_2_ fixation via the CBB cycle [66,67]. In this study, *Afipia carboxidovorans* were dominant species (Figure 2H; Supplementary Table S1). Interestingly, core functional rhizosphere cyanobacteria *Nostoc* sp. PCC7120 with significant differences were identified in the CH-F group (Figure 2J and Figure 3). Furthermore, metagenome-assembled genomes (MAGs) can identify host-associated novel species linked to specific biological processes via computational binning algorithms [68]. Among the 14 MAGs in this study, *Rhizobium tubonense* (Mu.14) and *Pseudomonas batumici* (Mu.15) were identified to be involved in the carbon fixation pathway, such as CBB, WL, and DC/4HB (Figure 6; Supplementary Table S7), implying their potential roles in carbon fixation.

### 4.3. Mycorrhizal Helper Bacteria Might Be Involved in the Growth of Cheilotheca humilis by Nitrogen Transformation Pathway

Increased evidence demonstrated that mycorrhizal fungi contributed to nitrogen acquisition by host plants [69]. In particular, the extraradical mycelium of mycorrhizal fungi could transfer nitrogen from the MHBs to the host plant [70]. Furthermore, MHBs can play roles in nitrogen fixation by utilizing nitrogenase [71]. For example, the *Rhizobium* genus was highly efficient in symbiotic nitrogen fixation [72,73,74]. In addition, nitrogen-fixing bacteria usually possessed a nif gene cluster (*nifB*, *nifH*, *nifD*, *nifK*, *nifE*, *nifN*, *nifX*, *hesA*, and *nifV*) [75]. In this study, the *Bradyrhizobium* genus, *Rhizobium* genus, and *Mesorhizobium* genus were identified to be dominant and core microorganisms (Figure 2 and Figure 3). In addition, the endophytic diazotroph *Herbaspirillum* genus improved the growth and nitrogen accumulation in plant [76,77]. Similarly, *H. rubrisubalbicans*, *H. seropedicae*, *H. huttiense*, and *H. hiltneri* were identified. In addition, MAGs analysis showed that these MHBs were mainly involved in assimilation, dissimilation, and denitrification (Figure 7; Supplementary Table S8), implying having key roles in the nitrogen cycle.

### 4.4. Mycorrhizal Helper Bacteria Might Be Involved in the Growth of Cheilotheca humilis by Phosphorus Metabolism Pathway

Phosphorus was of great significance for plant growth, yield, and survival. However, plants can hardly access it in most soils. Certainly, mycorrhizal symbiosis can enhance the phosphate uptake of the host plant [78]. Particularly, mycorrhizal fungi synergistically interacted with phosphate-solubilizing bacteria and transported it to their host plant [63]. It had been reported that *Enterobacter asburiae* promoted plant growth through the solubilization of various inorganic phosphates [79]. In this study, *Enterobacter asburiae* was identified to possess a higher abundance in the CH-B group (Figure 2J). Similarly, MAGs analysis showed that these MHBs were mainly involved in phosphorus solubilization (Figure 8; Supplementary Table S9), implying having key roles in phosphorus metabolism. In addition, a yam phyllosphere symbiont *Paraburkholderia* genus colonized the tomato phyllosphere and promoted plant growth by action of its ACC deaminase [80]. In this study, *Paraburkholderia phytofirmans* was defined as the core functional rhizosphere microbial genera, implying its key roles in phosphorus metabolism.

In summary, these findings in the myco-heterotrophic plant still provided novel insights into the interactions of the host plant, mycorrhizal fungus, and MHBs.

## Figures and Tables

**Table 1:** Details of classified MAGs recovered from *Cheilotheca humilis* metagenome.

MAG ID	Species Taxonomy	Closest Placement ANI	Completeness	Contamination	Contigs	Genome	N50	GC
			(%)	(%)	Num	Size		(%)
Mu.10	*UBA4124* sp.	79.31	81.24	0	29	804,524	44,130	41.84
Mu.21	*JABDFY01* sp.	77.59	96.4	2.94	136	2,157,904	23,608	57.59
Mu.16	*Aquitalea denitrificans*	90.99	97.86	0.43	63	4,193,559	106,226	58.73
Mu.37	N/A	N/A	77.53	3.04	216	1,326,563	7456	50.63
Mu.32	N/A	N/A	96.15	5.22	71	4,659,288	115,614	59.11
Mu.40	*JACAEB01* sp.	77.13	99.13	1.16	80	3,422,884	80,120	63.76
Mu.24	N/A	N/A	96.09	2.69	630	8,226,318	31,297	60.93
Mu.38	N/A	N/A	93.88	7.6	519	4,456,784	12,606	54.57
Mu.14	*Rhizobium tubonense*	84.43	89.81	3.27	455	5,778,709	23,353	59.38
Mu.8	N/A	N/A	91.88	2.78	606	5,927,924	13,162	46.75
Mu.15	*Pseudomonas_E batumici*	95.48	75.44	8.77	1698	6,777,919	4957	62.31
Mu.34	*Duganella* sp.	84.75	78.06	7.64	1233	5,166,334	4986	62.06
MU-F.10	*Roseiarcus*	79.98	74.25	6.28	1029	4,617,664	5534	62.82
MU-F.8	*Puia dinghuensis*	78.15	91.01	7.44	1585	6,948,697	5212	53.16

## Data Availability

The original contributions presented in this study are included in the article and Supplementary Material. Further inquiries can be directed to the corresponding author. The metagenome raw data has been submitted to the Sequence Read Archive at National Center for Biotechnology Information under BioProject ID: PRJNA1143161 (Accession number: SRR30134408-SRR30134413). The genome data of mycorrhizal fungus *Humicolopsis cephalosporioides* was deposited at GenBank under Bioproject ID: PRJNA1262328 (Accession number: JBPDIK000000000, https://www.ncbi.nlm.nih.gov/bioproject/PRJNA1262328/, accessed on 19 June 2025).

## Supplementary Materials

The following supporting information can be downloaded at: https://www.mdpi.com/article/10.3390/microorganisms13102387/s1, Figure S1: The isolated 66 endophytic microorganisms; Table S1: The top 20 phyla, family, genus, and species level of *Cheilotheca humilis* CH-B and CH-F; Table S2: Core taxa of rhizosphere microbial community in the *Cheilotheca humilis*; Table S3: KEGG and GO functional analysis of genes associated with *Cheilotheca humilis* rhizosphere metagenome; Table S4: The abundance of DE CCGs corresponding to the differential taxa in the *Cheilotheca humilis* rhizosphere metagenome; Table S5: The 55 metagenome-assembled genome (MAGs); Table S6: The identified 476 carbon cycling genes (CCGs) involving carbon fixation, carbon release, organic biosynthesis, organic degradation, organic transformation, and transporter modules by blast search against CCycDB; Table S7: The identified carbon cycle genes (CCGs) in 14 MAGs involving in-carbon fixation processes; Table S8:The identified nitrogen cycling genes (NCGs) in 14 MAGs involving in-nitrogen cycle processes; Table S9:The identified phosphorus cycling genes (PCGs) in 14 MAGs involving in phosphorus cycle process; Table S10: The isolated rhizospheric and endophytic microorganism.
