# Supplementary figures and images for: Metagenomics and In Vitro Growth-Promoting Experiments Revealed the Potential Roles of Mycorrhizal Fungus Humicolopsis cephalosporioides and Helper Bacteria in Cheilotheca humilis Growth
Source: Microorganisms. 2025 Oct 17;13(10):2387. doi: 10.3390/microorganisms13102387 (PMC12566112; doi:10.3390/microorganisms13102387)

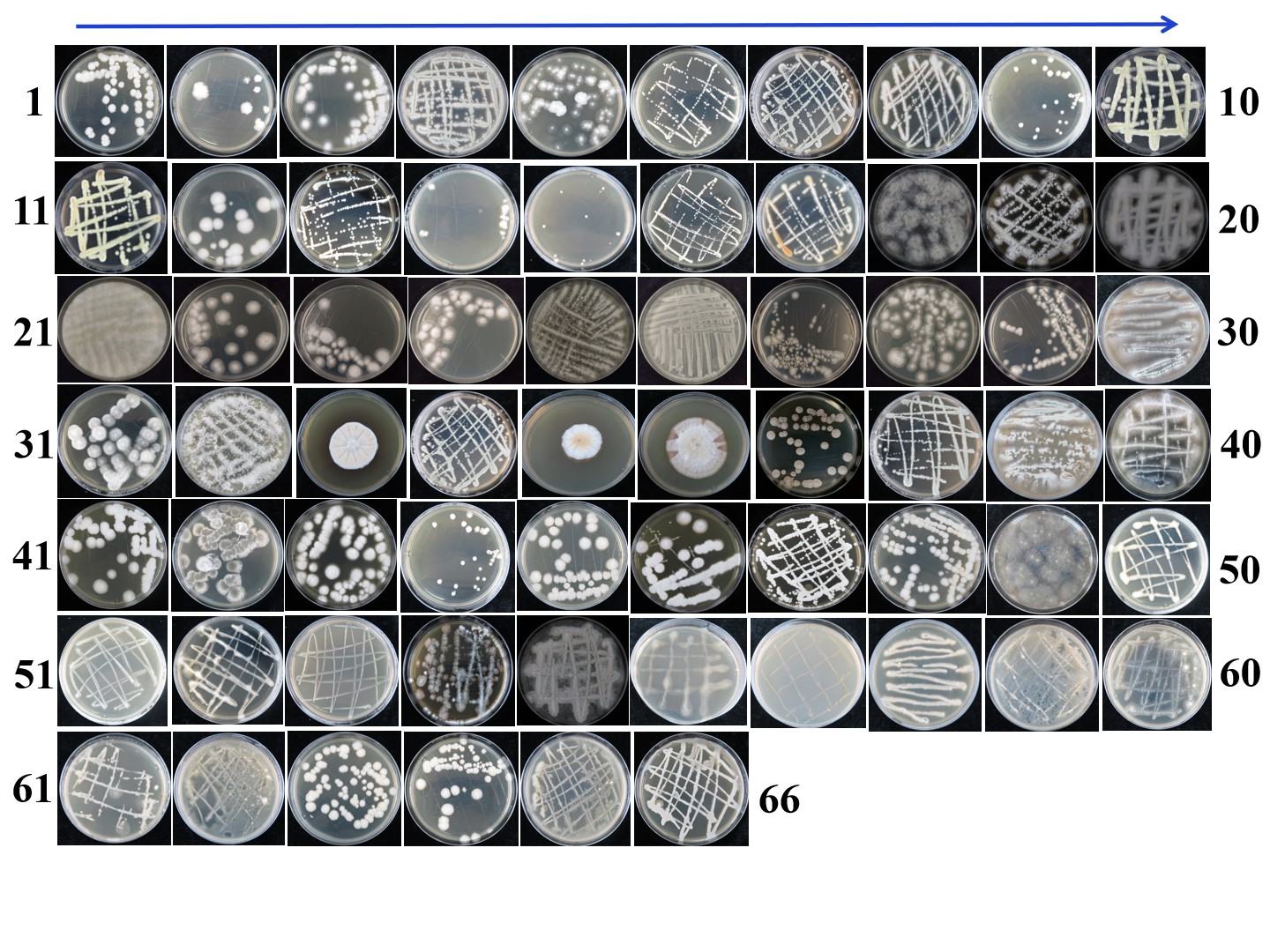

Supplement: Supplementary file 1 [file microorganisms-13-02387-s001.zip › Supplementary Figure S1.jpg]
